# Corporate Responses to Intimate Partner Violence

**DOI:** 10.1007/s10551-023-05461-6

**Published:** 2023-06-12

**Authors:** Layla Branicki, Senia Kalfa, Alison Pullen, Stephen Brammer

**Affiliations:** 1 Bath School of Management, University of Bath, Bath, BA2 7AY UK; 2 Macquarie Business School, Macquarie University, 4, Eastern Road, Macquarie Park, NSW 2113 Australia; 3 Bath School of Management, University of Bath, Bath, BA2 7AY UK

**Keywords:** Business ethics, Corporate social responsibility, Gender equality, Intimate partner violence, Stakeholder theory

## Abstract

Intimate partner violence (IPV) is among society’s most pernicious and impactful social issues, causing substantial harm to health and wellbeing, and impacting women’s employability, work performance, and career opportunity. Organizations play a vital role in addressing IPV, yet, in contrast to other employee- and gender-related social issues, very little is known regarding corporate responses to IPV. IPV responsiveness is a specific demonstration of corporate social responsibility and is central to advancing gender equity in organizations. In this paper, we draw upon unique data on the IPV policies and practices of 191 Australian listed corporations between 2016 and 2019, that collectively employ around 1.5 M employees. Providing the first large-scale empirical analysis of corporate IPV policies and practices, we theorise that listed corporations’ IPV responsiveness reflects institutional and stakeholder pressures which are multifaceted and central to corporate social responsibility. Our findings identify greater IPV responsiveness among larger corporations, as well as those corporations with higher proportions of women middle managers, greater financial resources, and more advanced employee consultation on gender issues. This paper concludes that there is a need for further research on corporate IPV responsiveness, to further illuminate corporate motivations, organizational support processes, and employee experiences.

## Introduction

Intimate partner violence (IPV) is among the most pernicious social issues facing global society today (Garcia-Moreno et al., 2006; Roy et al., 2019). The World Health Organization (WHO, 2013 p. vii) defines IPV as “behaviour by an intimate partner that causes physical, sexual, or psychological harm, including acts of physical aggression, sexual coercion, psychological abuse and controlling behaviours… [including] violence by both current and former spouses and other intimate partners.” Globally, approximately one third of women who have been in a relationship report that they have experienced some form of sexual or physical violence in their lifetime (WHO, 2017). IPV has significant economic and health impacts, including estimated economic costs of between 1% and 4% of global GDP (García-Moreno et al., 2015; Ribero & Sánchez, 2005), and multiple adverse impacts on women’s health (Ellsberg et al., 2008; Kapiga et al., 2017), including being the largest cause of women’s death by homicide (Carmichael et al., 2019; Devries et al., 2013). In Australia, the context for our study, PricewaterhouseCoopers (PwC, 2021) estimate that IPV costs the Australian economy $21.7 billion each year, and Women’s Safety New South Wales (2020) suggest that “one in four Australian women aged 18 to 44 years of age will experience domestic and family violence. This violence will be the single biggest preventable driver of their death, disability and illness.” A recent report by Australia’s National Research Organisation for Women’s Safety (ANROWS) (Morgan & Boxall, 2022) evidenced that domestic violence escalated during the pandemic, especially for those women who lost their job, taken a pay cut or had their hours reduced.

Gender equity remains the “unfinished business of our time… [and] the greatest human rights challenge in our world” (United Nations, 2020). Responsibility for human rights and gender equity is a moral obligation of institutions including corporations with the advancement of gender equity, and the responsible management of gendered violence, forming part of corporate responsibility (de Jonge, 2018). A listed corporation is “not merely an economic actor, but also a social and political actor… no societal concern is unrelated to business, whether in its antecedents or consequences, or in its very constitution, and domestic violence cannot be and should not be an exception” (Wilcox et al., 2021, p. 7). Even though IPV affects not only women (Wathen et al., 2018), it is women that most frequently experience IPV at home and work (Johnson, 2011).

Promoting equality, inclusion, non-discrimination, and representation of women in economy and society has contributed to the development of a significant literature examining gender equity in organizations (see, Grosser, 2009; Grosser & Moon, 2019). Research has explored gender issues in relation to employment (Showalter, et al., 2021), organization boards (Bear et al., 2010), workplace practices (Grosser & Moon, 2005; Larrieta-Rubın de Celis et al., 2015), supply chains (Barrientos et al., 2003; Prieto-Carrón, 2008), and community impacts and involvement (Keenan et al., 2014; McCarthy, 2017).

However, while research has significantly advanced both the analysis of corporate responsibilities in relation to gender (e.g., Karam & Jamali, 2013), and begun to apply feminist theory into considerations of listed corporations’ responsibilities (Grosser et al., 2017; McCarthy, 2018), relatively little is known about the specific IPV resources and supports available to employees in their workplaces(MacGregor et al., 2017). Research has examined the relationship between IPV and the workplace (Al-Modallal et al., 2016; Deen et al., 2022; Lloyd & Taluc, 1999; Shepard & Pence, 1988; Wilcox et al., 2021), the lived experience of employees suffering IPV (MacGregor et al., 2016), workplace resourcing and support for employees experiencing IPV (MacGregor et al., 2017), and the role of trade unions in supporting victims of domestic violence in the workplace (Wibberley et al., 2018). Although IPV has been the subject of strands of research in industrial relations (e.g Baird et al., 2014; Boxall et al., 2020; Weatherall et al., 2021), human resource management (HRM) (e.g., Showalter et al., 2021; Tolentino et al., 2017), and organizational behaviour (e.g. Deen et al., 2021; Garcia et al., 2017), relatively little business ethics research has addressed IPV, as noted in the special issue call for papers. An exception is de Jonge’s paper (2018) which discusses the integration of IPV within corporations’ social responsibilities, noting the expanding scope of social responsibilities over time, and highlighting that IPV responsiveness can be conceptualized through rights-based, social contract, and feminist perspectives.

In this paper, how listed corporations are addressing IPV through programs, policies, and initiatives that support employees experiencing IPV are examined. Specifically, we conceptualize corporate IPV responsiveness as a specific demonstration of corporate social responsibility and draw upon institutional and stakeholder theories to develop hypotheses regarding the influences on the extent of listed corporations’ IPV responsiveness. We test our hypotheses using longitudinal quantitative data on the measures taken by Australian listed corporations to address employee experiences of IPV. In particular, hand coded data on IPV support initiatives extracted from Australia’s Workplace Gender Equality Agency (WGEA) is used to provide unique insights regarding the influences on corporate policies and support mechanisms among Australian listed corporations for the period 2016–2019. We ask: (1) What institutional, stakeholder, and organizational factors explain variations across listed corporations in the extent of their IPV responsiveness? (2) What are the implications of our findings for policy and practice?

The paper makes two main contributions. First, we provide the first comprehensive empirical analysis of corporate IPV responsiveness, highlighting how corporations’ institutional and stakeholder environments shape their IPV responsiveness. Second, we build upon our empirical analysis to theorize corporate responses to IPV to suggest how policy and practice in relation to IPV might be improved to ensure that IPV is integrated within corporations’ social responsibilities.

## Intimate Partner Violence and Work

A significant body of research examines IPV as a workplace issue (see MacGregor et al., 2021; Deen et al., 2022; O’Leary-Kelly et al., 2008), suggesting that IPV is a growing concern for the responsibilities of businesses. Research has shown that workplaces are valuable sources of support for employees experiencing IPV (Reeves and O’Leary‐Kelly, 2007; MacGregor et al., 2021, 2022). MacGregor et al.’s (2022) review of existing research reinforces how important work and employment is to employees experiencing IPV, including the financial benefits of work, workplace support, work acting as an escape from IPV, and work being a safe place away from violence (though it is important to acknowledge that violence often follows people into work). Maintaining employment provides opportunities to maintain meaningful social connections (Wilcox et al., 2021) and more importantly economic independence and stability (Showalter et al., 2021), which assists women to leave violent relationships. Whilst a variety of workplace support mechanisms are available for employees experiencing IPV, awareness and recognition of such support varies considerably across employees (MacGregor et al., 2017). Similarly, employers vary in their recognition of the complexities surrounding the experiences of IPV and the need for flexibility in approaches when they respond to employees’ request for support (e.g., Samuel et al., 2011).

The negative effects of IPV on work are also well documented (see, MacGregor et al., 2021 for review, and Al-Modallal et al., 2016; Lloyd & Taluc, 1999; Shepard & Pence, 1988; Węziak-Białowolska et al., 2020). Laharnar et al., (2015, p. 110) note that the consequences of IPV on work include the “abuser using work-interference tactics (harassing co-workers, affecting employee’s ability to get to work, stay at work), survivor absenteeism due to illness, injury or mental health and reduced job performance and productivity due to employee’s difficulty concentrating and absence.” To illustrate, the impact of IPV on women’s employment can be evidenced in the case of a pharmaceutical sales representative who,

“… in 2010 contacted her employer of fourteen years to report that police advised her to go into hiding after her ex-husband, against whom she had a no-contact order, held a gun to her and threatened to kill her in a crowded restaurant. While her employer gave her thirteen weeks of unpaid leave under its general leave policy, it did not guarantee her future employment or assist her in any other way. When her leave expired, her employer informed her that she had sixty days to find and apply for another open position for which she had to compete with the general public. Because her ex-husband’s criminal prosecution was still pending, Ms. Goodson informed her employer that it was still not safe for her to return to work. The company ultimately terminated her employment when she did not return to work for six months” (Arnott & Hobday, 2014, p. 1-2).

Of concern, work interference—lateness, absence, reduced performance (Al-Modallal et al., 2016)—can trigger disciplinary sanctions from an employer, particularly when IPV has not been disclosed (Wibberley et al., 2018). Therefore, it is not surprising that research reports that employees are often reluctant to disclose IPV (Al-Modallal et al., 2016). For example, Swanberg and Logan (2005, p. 6) found that IPV disclosures were often “forced” because of safety concerns, suspicions from managers or co-workers, or because the abuser “shows up at work.”

The reluctance of employees to disclose IPV to employers therefore inhibits organizations’ ability to effectively support employees (Laharnar et al., 2015; MacGregor et al., 2021; Swanberg et al., 2007; Swanberg & Logan, 2005). Existing research suggests that employees experiencing IPV are apprehensive about the recipient of the disclosure responding negatively (see, Woerner et al., 2019), fearful about a “lack of support at work|” (MacGregor et al., 2022, p. 12), and anxious about the confidentiality of their IPV disclosure (Laharnar et al., 2015; MacGregor et al., 2022; Woerner et al., 2019). Swanberg and Logan (2005) suggest that employees decide not to disclose IPV when they fear their job will be at risk, experience shame, or feel that they can handle the situation independently. Consistent with concerns regarding reluctance to disclose IPV, attitudes that sensitize employees to IPV are a key factor in shaping effective prevention strategies and policies (Gracia et al., 2020). Given the complexities associated with IPV disclosure, it is therefore unlikely that there is a “one size fits all” solution to organizational IPV responsiveness (MacGregor et al., 2022, p. 14).

Even though IPV is consequential for organizations in that reduced productivity among employees affected by IPV bears economic consequences (de Jonge, 2018), the most profound impacts of IPV fall upon women affected by IPV as their economic independence and social agency are threatened (O’Leary-Kelly et al., 2008; Deen et al., 2022). In this sense, corporate IPV responsiveness has the potential to advance gender justice substantially, enhancing the lives and livelihoods of women. Significantly, organizations are increasingly subject to legal responsibilities to address IPV. According to Safe Work Australia, family violence can be a work health and safety issue when the perpetrator makes threats or carries out violence on a family member while they are at work, including if an employee’s workplace is their home. This is now supported by case law (see, Workers Compensation Nominal Insurer v Hill [2020] NSWCA 54) following the tragic death of Michel Carroll at the hands of her de-facto partner and colleague Steven Leslie Hill.

## Institutional and Stakeholder Influences on Corporate Intimate Partner Violence Responsiveness

**Fig. 1:**
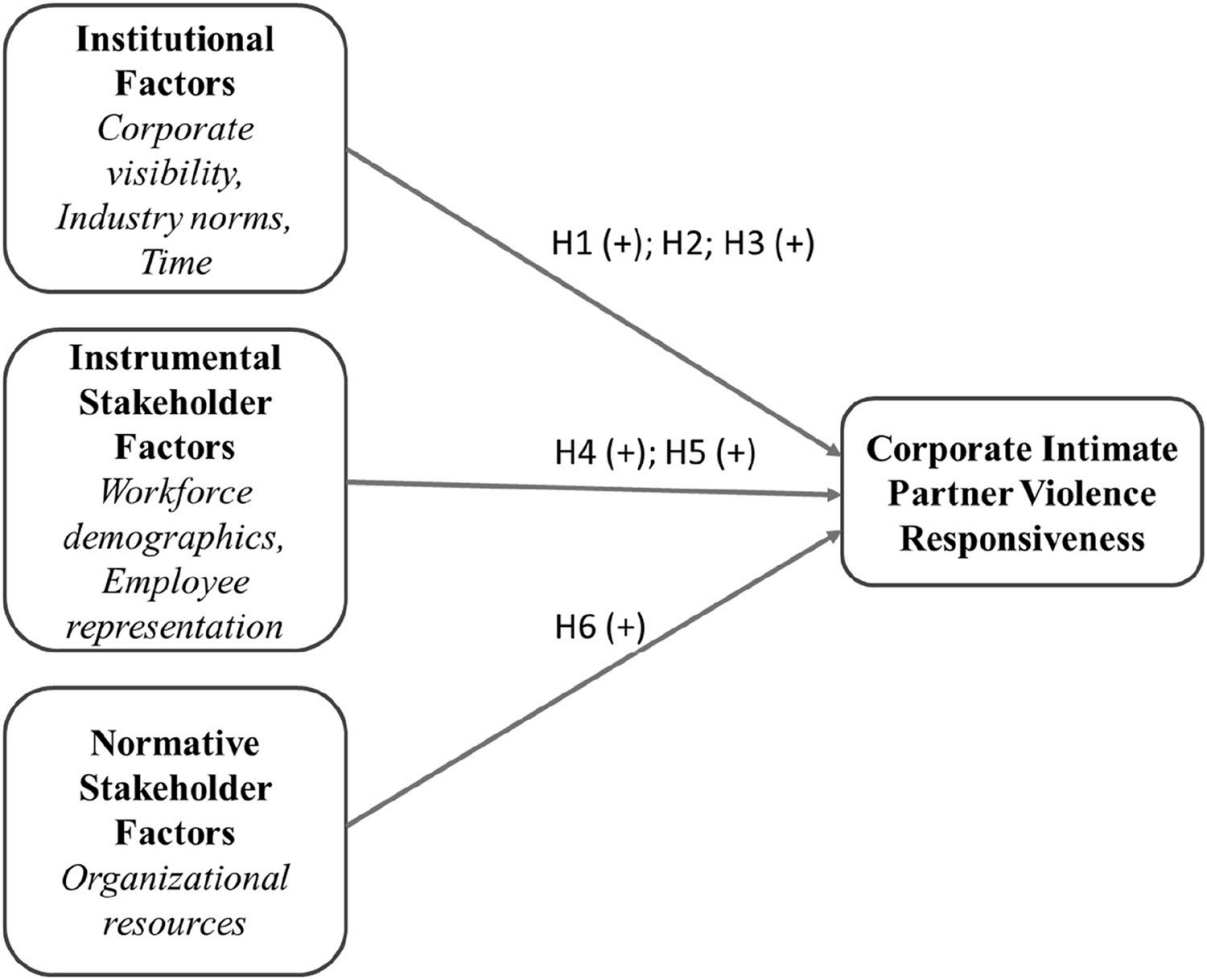
Institutional and stakeholder influences on IPV responsiveness

In this paper, consistent with a large and growing literature focusing on corporate social responsibility towards employees (Jones et al., 2019; Glavas, 2016; Voegtlin & Greenwood, 2016), corporate IPV responsiveness is theorized as an element of corporate social responsibility. CSR reflects “the notion that corporations have an obligation to constituent groups in society other than stockholders and beyond that prescribed by law and union contract” (Jones, 1980, p. 59–60). Our conceptual model is described in Fig. 1, below. Figure 1 builds upon two approaches to theorizing listed corporations’ social responsiveness that have attracted particular attention in the last two decades: (i) institutional theory (Campbell, 2007), and (ii) stakeholder theory (Donaldson & Preston, 1995; Laplume et al., 2008), by identifying three clusters of influences on IPV responsiveness. The first cluster of influences reflect institutional pressures on listed corporations, especially the distinctive pressures that apply to very large corporations, norms regarding IPV responsiveness that vary across industry sectors, and increasing societal expectations of IPV responsiveness over time. The second cluster of influences reflect instrumental stakeholder pressures faced by listed corporations in relation to IPV responsiveness. These pressures reflect potential benefits to corporations from responding to concerns among employees and their representatives for improved responsiveness towards employees that experience IPV. The third cluster of influences reflect normative influences on IPV responsiveness, especially the importance of discretionary financial resources that enable corporations to respond to IPV concerns among employees and within wider society. We theorize that listed corporations’ overall IPV responsiveness will reflect the extent to which they experience these institutional, stakeholder and normative pressures. Even though these motivations are, in practice, enmeshed with each other in shaping IPV responsiveness, it is nevertheless useful to distinguish between their specific implications for likely patterns of IPV responsiveness.

### Institutional Influences on IPV Responsiveness

Institutional factors play an important role in shaping CSR activities (Husted & Allen, 2006; Jamali & Carroll, 2017). Institutional theory proposes that organizational success and survival depend critically upon compliance with social norms of acceptable behaviour (DiMaggio & Powell, 1983). DiMaggio and Powell (1983) suggest that organizations within a given institutional field tend to develop similar responses to institutional pressures to maintain their legitimacy. Institutional pressures take various forms, including coercive, mimetic, and normative pressures (DiMaggio & Powell, 1983; Scott, 2005). Institutional pressures shape whether organizations’ behaviours are, or are not, legitimate in the sense that they satisfy “a generalized perception or assumption that the actions of an entity are desirable, proper, or appropriate within some socially constructed system of norms, values, beliefs, and definitions” (Suchman, 1995, p. 574). Legitimate corporate behaviour varies both across contexts, and within contexts over time, as societal expectations of appropriate conduct evolve because of shifting societal priorities and considerations. For example, the evolving social contract between employees and employers contributed to shifts in perceptions of the role of corporations in relation to corporate responsibilities (Westermann-Behaylo et al., 2014).

How might institutional pressures shape listed corporations’ IPV responsiveness? First, prior research has shown that corporations are differentially responsive to institutional pressures (Oliver, 1991). Larger corporations have been shown to be particularly responsive to institutional pressures (Hess & Warren, 2008) because they face greater scrutiny from government regulatory bodies and the media (Goodstein, 1994; Powell, 1991). Large, highly visible, corporations are also increasingly subject to pressure from activists, partly because their actions, strategies and their outcomes are more accessible to the public (Chiu & Sharfman, 2011). Pressures applied from the public tend to lead the very large corporations that are listed on the stock exchange to perceive themselves as subject to greater institutional pressure via a form of “implicit regulation” that directs such corporations to adopt higher standards of behaviour than others because they fear social sanction.

Second, many institutional accounts define industry sectors as distinct institutional fields because industry sectors tend to be characterized by a shared system of rules, beliefs, and norms that serve to distinguish one field from another (DiMaggio & Powell, 1983). Corporations pay particularly close attention to competitors within their industry and are likely to be more sensitive to information about the adoption of new practices by peers within their industry than those in other sectors (Rao & Sivakumar, 1999). Corporations not only actively seek information and insight regarding the actions of their industry peers, but also exhibit a tendency to imitate practices used by their competitors even when evidence regarding the success of these practices is absent (DiMaggio & Powell, 1983). Prior research has shown that CSR activities are heavily shaped by the pressures in corporations’ industry environments, reflecting distinctive opportunities and challenges in relation to social and environmental issues (Ali et al., 2017; Campbell, 2007). Thus, it is probable that corporations’ engagement in specific forms of CSR is significantly shaped by the extent to which industry peers are also addressing the same issue, especially for an emergent domain of CSR such as IPV responsiveness.

Third, given the growing salience of IPV in societal discourses in recent years, pressures on listed corporations to be seen as responding legitimately to IPV issues has likely intensified (see, MacGregor et al., 2017), inferring that expectations about IPV responsiveness might be growing over time. Particularly in Australia, the social salience of IPV generally, and the attention to IPV as a workplace issue specifically, has been rapidly growing in recent years. Several high-profile and highly distressing case examples of IPV which were related to work and involved the death of the victim, have led to growing recognition of the importance of employer responsiveness to IPV (Hilton, 2020). Legal frameworks in Australia prescribe minimum employer responsibilities in relation to supporting employees experiencing IPV. For example, employees experiencing IPV can take unpaid family and domestic violence leave, request flexible working arrangements, and take paid or unpaid personal/carer leave, in certain circumstances (Fair Work Ombudsman, 2020). Additionally, employers must take reasonable practical steps to keep any information about an employee’s IPV situation confidential. Reflecting these arguments, we hypothesize that:

#### Hypothesis 1:

There is a positive relationship between corporation size and IPV responsiveness.

#### Hypothesis 2:

There is significant variation across industry sectors in IPV responsiveness.

#### Hypothesis 3:

IPV responsiveness is increasing over time, reflecting the growing societal salience of IPV issues.

### Instrumental Stakeholder Influences on IPV Responsiveness

Stakeholder perspectives on corporations’ socially responsive behaviours have tended to emphasize the “business case” for addressing social issues (Carroll & Shabana, 2010). There is a long history of corporate employee responsiveness underpinned by the benefits that come in the form of employee attraction, recruitment and retention (Bhattacharya et al., 2008; Greening & Turban, 2000), employee identification (Kim et al., 2010; Rodrigo & Arenas, 2008), and employee commitment (Brammer et al., 2007; Peterson, 2004). Employees, and their concerns, are clearly recognized as being of enduring importance for organizations (Clarkson, 1995; Phillips, 2003). Pava and Krausz (1997, p. 346), aligning with Porter and Kramer’s (2002) conception of shared value creation, note that often “a sensitivity to employee needs is both good business and represents socially responsible behavior” (Pava & Krausz, 1997, p. 346) although they do argue that corporations need to be mindful of understanding where and when CSR comes at a cost to financial performance.

What might an instrumental stakeholder approach to IPV responsiveness imply for which organizations are likely to exhibit greater responsiveness? An instrumental approach would predict that IPV responsiveness would likely be greater among listed corporations where employee satisfaction plays an especially important role in organizational success. Larger corporations that employ more employees have significant financial opportunities to reduce the costs of labour turnover, training, and recruitment by being highly responsive to employees’ needs (Du et al., 2015). It is also expected that there are significant differences across industry sectors, for example in service-sector organizations where prior research has indicated that there is a strong relationship between employee satisfaction and customer satisfaction (Bitner, 1995; Hogreve et al., 2017). A second category of corporations that have significant benefits to earn from satisfied employees are those in highly innovative sectors where employee creativity and innovative capacity play an important role in shaping competitive advantages (Brammer et al., 2015).

Perhaps most important, though, are direct pressures within corporations to provide better support to employees that experience IPV. Female employees are known to play an important role in encouraging organizations to adopt more pro-social policies in general, and more employee-oriented policies and practices (Dobbin et al., 2011). Dobbin et al., (2011, p. 388) note that “women were most often identified as diversity program champions in interviews we conducted with human resources (HR) managers in 2008 and 2009 at 64 workplaces in four large cities”. Therefore, where a higher proportion of a listed corporation’s employees are female, we expect greater responsiveness to IPV because managers, especially those with access to senior management contexts, have a capacity to play a greater role in shaping the development and implementation of corporate policies.

In addition to the importance of differences in the prevalence and representation of women within corporations for IPV responsiveness, the presence of formal consultative processes is also likely to be significant. Although research has suggested an ambiguous orientation, especially historically, between trade unions and gender equity (Kirton, 2019), there is a growing literature that emphasizes the importance of formal employee involvement and consultation in achieving a range of workplace outcomes (Dibben et al., 2011; Timming, 2012). Employee consultation and involvement in relation to gender equity has been found to play a vital role in maintaining high levels of commitment and engagement among female employees (Daly et al. 2008; Gilbert et al., 1999). Formal consultation on gender issues encourages employee commitment because it signals equal career opportunity and the possibility of work–family integration, leading employees to reciprocate organizations’ goodwill by demonstrating commitment and reduced turnover intentions, consistent with social exchange theory (Blau, 1964). Thus, consultation provides the means for employers to understand employee concerns, and a mechanism to reinforce employee commitment by demonstrating organizations’ intention to address gender issues.

#### Hypothesis 4:

There is a positive relationship between the percentage of women in corporations’ workforce, in management positions, and on corporations’ boards and IPV responsiveness.

#### Hypothesis 5:

There is a positive relationship between formal gender equity consultation and IPV responsiveness.

### Discretionary Influences on IPV Responsiveness

Even though considerable stakeholder theorizing regarding CSR has adopted an instrumental perspective, stakeholder thinking has always retained a prominent normative orientation (Donaldson & Preston, 1995). In their critique of instrumental conceptualizations of both corporate social responsibility and stakeholder theory, Freeman and Liedtka (1991) articulate the proposition that “corporations are places where both individual human beings and human communities engage in caring activities that are aimed at mutual support and unparalleled human achievement” (Freeman & Liedtka, 1991, p. 96). Interest in normative considerations of how organizations might approach stakeholder relationships has flourished in recent years (e.g., Alacovska & Bissonnette, 2021; Carmeli et al., 2017). Prior research (Liedtka, 1996; Wicks et al., 1994) conceptualizes caring in organizations as reflecting an organization’s ‘deep structure’ of values and organizing principles centered on fulfilling employees’ needs, promoting employees' best interests, and valuing employees' contributions” (McAllister & Bigley, 2002, p. 895). A caring approach reflects policies, practices, and behaviors that reflect a “firm’s intent to ‘go the extra mile’ in treating its employees well” (Bammens, 2016, p. 246).

What would a normative stakeholder perspective imply for organizational IPV responsiveness? A normative stakeholder perspective reflects the needs of organizational members as they arise in practice. Normative motivations for IPV responsiveness resonate with Pava and Krausz’s (1997) suggestion that “local knowledge” can play an important role in shaping corporations’ responsibilities in particular situations. “Local knowledge” encompasses the idea that to “design effective programs to satisfy social responsibilities, the organization must possess knowledge about the specific social problem” (Pava & Krausz, 1997, p. 339). This criterion couples a sense of awareness of an issue, with the capacity, derived from a corporation’s resources and capabilities, to address the issue in a relatively effective way. Regarding corporations’ capacity to respond to employees’ IPV needs, prior research has demonstrated that organizational responses to external pressures are dependent upon their resource profile (Bourgeois, 1981; Cyert & March, 1963). Resources endow corporations with latitude in relation to determining their response to normative pressures. Organizational slack “is that cushion of actual or potential resources which allows an organization to adapt successfully to internal pressures for adjustment or to external pressures for change in policy, as well as to initiate changes in strategy with respect to the external environment” (Bourgeois, 1981, p. 30). Higher levels of resources provide corporations with greater flexibility toward, and better understanding of, external influences (Cyert & March, 1963). Slack enhances an organization’s discretionary ability to resource care-based concern for employees, and thus might encourage greater IPV responsiveness.

#### Hypothesis 6:

There is a positive relationship between availability of financial resources and IPV responsiveness*.*

## Methods

### Study Context and Sample

Australia is the context for our study because of the salience of IPV in society, including work organizations (Workplace Safety NSW, 2020; Domestic Violence Work Aware, 2020), and the availability of unique data regarding organisational support for employees experiencing IPV. Our data collection starts with *Australia’s Workplace Gender Equality Agency* (WGEA) database, an Australian Government statutory agency created by the Workplace Gender Equality Act, 2012 and tasked with promoting gender equality in Australian workplaces. Australian Government policy is that public sector information collected by public agencies—such as that contained in the WGEA database—is a national resource and that to the maximum extent possible such data should be made publicly available. As a result, WGEA developed a Data Explorer which enables public access to organizational responses to mandatory and voluntary reporting made annually.^1^ The Workplace Gender Equity Act (2012) seeks to (i) promote and improve gender equality (including equal remuneration between women and men) in employment and in the workplace, (ii) support employers to remove barriers to the full and equal participation of women in the workforce, (iii) promote, amongst employers, the elimination of discrimination on the basis of gender in relation to employment matters (including in relation to family and caring responsibilities), (iv) foster workplace consultation between employers and employees on issues concerning gender equality in employment and in the workplace, and (v) improve the productivity and competitiveness of Australian business through the advancement of gender equality in employment and in the workplace (Workplace Gender Equality Act, 2012).

WGEA promotes workplace equality in various ways, including by providing advice, practical tools, and education, but also by generating reporting data through an annual survey which informs benchmarking reports and publicly accessible data regarding organisations’ policies and actions on gender equity. Under the Act, non-public sector organisations with more than 100 employees are required to report annually against six gender equality indicators including the representation of women in leadership, equal remuneration between women and men and policies and actions they are taking in respect of these gender equality indicators. Additionally, WGEA operate a voluntary reporting programme that enables public and third-sector organizations to report and benchmark their gender equity progress. This legislation has resulted in the collection of a unique and extensive data set that encompasses more than 4.3 million employees—which equates to more than 40% of the Australian workforce. To maintain comparability of organizational types in our analysis, and to facilitate the matching of WGEA data with financial data, we focused on corporations listed on the Australian Stock Exchange from 2016 to 2019, for which financial and other data are relatively available and which share financial, governance, and regulatory similarities. Our sample comprises all listed Australian companies focusing on the availability of both WGEA data and Thompson-Reuters financial data. Matching WGEA data and financial data from Thomson-Reuters DataStream leads to an unbalanced panel dataset of 682 corporation/years, relating to 191 specific listed corporations, each contributing on average 3.6 years of data (out of a possible 4 years) to the overall sample. These 191 firms collectively constitute over 90% of the capitalization of the Australian Stock Exchange. In total, our sample corporations employ between 1.47 M and 1.54 M employees in the years studied, representing around 36% of all the employees in the WGEA database.

### Dependent Variable—Corporate IPV Responsiveness

Among the data collected in the WGEA survey are organisational policies and practices on IPV. Since 2016 WGEA has collected data that captures: (i) whether organizations have a formal policy or strategy in place regarding support for employees who are experiencing IPV, (ii) a summary indicator that describes whether organizations provide any support measures to employees experiencing IPV, and (iii) whether they have any of 15 specific measures in place to support employees that encounter IPV. Specific measures addressed in the survey include training, referral to support services, domestic violence leave arrangements, flexible work arrangements, changes of office/workplace location, and financial assistance—a full list of the measures used in the survey are provided in Table 1. In each case, organizations are asked: “Other than a formal policy and/or formal strategy, do you have the following support mechanisms in place to support employees who are experiencing family or domestic violence?” Respondents are required to provide Yes/No answers.^2^ Together these 17 indicators (1 indicator of formal policy/strategy, 1 summary support indicator, 15 specific policies/support measures) provide a unique insight into organisational activities to support employees experiencing IPV.

**Table 1:** Variation in IPV policies and programs across industry sectors

		Mining & Construction	Manufacturing	Transportation, Communication, & Utilities	Wholesale & Retail trade	Finance, Insurance, & Real estate	Services	All sectors
		(N = 134)	(N = 145)	(N = 81)	(N = 78)	(N = 88)	(N = 156)	(N = 682)
Has a formal policy or formal strategy	No. of observations with policy/program	74	80	53	35	60	91	393
	As a % of observations in sector	55.2%	55.2%	65.4%	44.9%	68.2%	58.3%	57.6%
Has some measure in place to support employees	No. of observations with policy/program	130	137	81	73	84	143	648
	As a % of observations in sector	97.0%	94.5%	100.0%	93.6%	95.5%	91.7%	95.0%
Employee assistance program	No. of observations with policy/program	127	136	81	65	84	139	632
	As a % of observations in sector	94.8%	93.8%	100.0%	83.3%	95.5%	89.1%	92.7%
HR or other staff training	No. of observations with policy/program	33	16	29	10	22	23	133
	As a % of observations in sector	24.6%	11.0%	35.8%	12.8%	25.0%	14.7%	19.5%
Referral to support services	No. of observations with policy/program	54	47	47	41	52	64	305
	As a % of observations in sector	40.3%	32.4%	58.0%	52.6%	59.1%	41.0%	44.7%
Access to any leave (overall measure)	No. of observations with policy/program	110	118	74	57	69	129	557
	As a % of observations in sector	82.1%	81.4%	91.4%	73.1%	78.4%	82.7%	81.7%
Paid domestic violence leave	No. of observations with policy/program	33	34	42	19	23	43	194
	As a % of observations in sector	24.6%	23.4%	51.9%	24.4%	26.1%	27.6%	28.4%
Unpaid domestic violence leave or unpaid leave	No. of observations with policy/program	110	113	72	57	69	129	550
	As a % of observations in sector	82.1%	77.9%	88.9%	73.1%	78.4%	82.7%	80.6%
Domestic violence clause in an enterprise or workplace agreement	No. of observations with policy/program	7	16	11	11	0	18	63
	As a % of observations in sector	5.2%	11.0%	13.6%	14.1%	0.0%	11.5%	9.2%
Workplace safety planning	No. of observations with policy/program	19	23	17	14	19	25	117
	As a % of observations in sector	14.2%	15.9%	21.0%	17.9%	21.6%	16.0%	17.2%
Confidentiality of disclosure	No. of observations with policy/program	81	78	73	54	68	117	471
	As a % of observations in sector	60.4%	53.8%	90.1%	69.2%	77.3%	75.0%	69.1%
Protection from adverse action or discrimination	No. of observations with policy/program	58	56	50	23	55	82	324
	As a % of observations in sector	43.3%	38.6%	61.7%	29.5%	62.5%	52.6%	47.5%
Flexible working arrangements	No. of observations with policy/program	97	103	72	56	74	129	531
	As a % of observations in sector	72.4%	71.0%	88.9%	71.8%	84.1%	82.7%	77.9%
Financial support	No. of observations with policy/program	44	18	19	21	33	48	183
	As a % of observations in sector	32.8%	12.4%	23.5%	26.9%	37.5%	30.8%	26.8%
Change of office location	No. of observations with policy/program	38	25	41	38	32	52	226
	As a % of observations in sector	28.4%	17.2%	50.6%	48.7%	36.4%	33.3%	33.1%
Emergency accommodation assistance	No. of observations with policy/program	18	10	17	3	9	19	76
	As a % of observations in sector	13.4%	6.9%	21.0%	3.8%	10.2%	12.2%	11.1%
Medical services	No. of observations with policy/program	40	29	16	9	11	27	132
	As a % of observations in sector	29.9%	20.0%	19.8%	11.5%	12.5%	17.3%	19.4%

For each listed corporation/year, we hand coded data from the WGEA data explorer to create a set of 17 dummy variables, each taking the value of 1 if a given policy or support measure is in place, and zero otherwise. In our descriptive analysis, patterns in each individual indicator across industries were analysed. We also sum across these 17 indicators to provide an overall measure of each listed corporation’s responsiveness on IPV to investigate changes in their overall frequencies over time. Because the summary variable that captures whether any form of employee support is available overlaps with the individual indicators, we excluded it from the calculation of our dependent variable for regression analysis, which is the sum of the policy/strategy indicator and the 15 individual support indicators. We draw upon four annual reporting cycles of the WGEA data from 2016 to 2019 inclusive, thus affording our analysis an opportunity to observe changing IPV responsiveness over this period.

### Independent Variables

To conduct our analyses, we link data from WGEA’s data collection on IPV responsiveness to other listed corporation data, some of which was separately hand-collected from other areas of the WGEA database, and some of which was collected from Thomson-Reuters DataStream, a leading source of financial and accounting data. In addition to providing information on IPV, WGEA report data on a range of organizational characteristics that are likely to shape their responsiveness to employees experiencing IPV.

First, WGEA collect data on the workforce composition in each organisation at various levels of seniority—specifically, data were extracted on the overall % women in a corporation’s workforce, as well as the % of women in managerial positions, the % of women in senior managerial positions, and the % of women on a corporation’s board of directors. Industrial relations in the Australian context are such that employer-employee relations in many workplaces is governed by a workplace or enterprise agreement. These agreements are periodically negotiated between employers and employee representatives (often trade unions) and codify commitments regarding a wide range of workplace issues and concerns. The presence or absence of these formal consultative processes provides signals pertaining to the nature of employee-representative dialogue mechanisms. Reflecting this, we also extracted two variables from the WGEA database that capture the nature of workforce consultation and representation: a binary variable that takes a value of 1 if some or all salaries at a corporation are established through establishment agreements, and zero otherwise; and a binary variable that takes a value of 1 if there has been formal consultation with employees/their representatives on gender equity issues, and zero otherwise.

Financial data were obtained from Thomson-Reuters DataStream. Corporation size was captured by the natural logarithm of the number of employees, and visibility to stakeholders through membership of the ASX50 index, the largest and most visible listed corporations in Australia. The financial resource environment of corporations was captured through three variables: return on total-assets, to capture profitability, long term debt as a % of total assets, and cash as a % of total assets. These measures are well established in the literature on organizational slack (Bourgeois, 1981). Each corporation’s principal industry sector was obtained as classified according to the standard industrial code system.

### Data Analysis

To facilitate the analysis, data on each corporation/year were combined into an Excel sheet, which was imported to IBM SPSS Statistics 25.0 for analysis. Our analysis took two main forms— (a) a descriptive overview of the variation in the prevalence of IPV policies and practices across industry sectors and time, shedding light on the aggregate level and nature of IPV responsiveness among Australian listed corporations; (b) regression analysis that explores the relationships between the characteristics of listed corporations and IPV responsiveness. Regarding (b), tests indicate that the dependent variable for our regression modelling—the count of the IPV policy/practice areas supported by a given corporation each year—is normally distributed. This enabled us to model the influences on IPV responses using Ordinary Least Squares regression analysis. To improve causal inferences, all time-variant independent variables are lagged by one year relative to the dependent variable.

## Findings

### Comparing IPV Policies and Practices Across Industries and Over Time

We start with a descriptive analysis of the extent and nature of IPV policies and programs and how these are evolving over time, before turning our attention to modelling the factors associated with corporate IPV responsiveness—as captured by the count of a corporation’s policies and programs present each year—to test our hypotheses.

Table 1 provides an overview of how common IPV support programs and policies are among Australian listed corporations, pooled over the four-year range in our data but broken down by industry sector. Overall, around 58% of listed corporations have a formal policy or strategy regarding support for employees experiencing IPV, and 95% of listed corporations offer some form of support to those employees. Formal policies or strategies are more commonly found in the finance and transport/ communication sectors (68% and 65% respectively), than they are in mining (55%), wholesale/retail (45%), manufacturing (55%) and other services sectors (58%). These differences suggest that a corporations’ industry environment plays a significant role in shaping available IPV support initiatives. By far the most commonly available measures taken by listed corporations to support employees experiencing IPV concern confidential disclosure, flexible work arrangements and allowing access to leave, most often unpaid. While approximately four-fifths of listed corporations offer leave and flexible working, and about 70% offer confidential disclosure, it is striking that beyond those measures the proportion of listed corporations implementing a given policy or program is much lower, typically being present in fewer than 40% of cases. For example, around 50% of listed corporations offer internal protection to employees experiencing IPV from discrimination, and around 45% offer to refer employees to support services. A little less than a fifth of corporations offer training to staff on IPV, or other workplace safety training. Other support programmes are relatively rarely available—for example, only 20% of listed corporations offer access to medical services, 11% offer access to emergency accommodation, 27% offer financial support, and 33% offer a change of workplace location. Overall, these patterns possibly signal a relatively reactionary and/or tokenistic approach to IPV support across many corporations.

The analysis demonstrates that while most listed corporations have some high-level policy commitment and offer some form of support to employees experiencing IPV, relatively few have deep and well-resourced IPV commitments that go beyond unpaid leave and flexible working. Looking at industry differences, these appear to be substantial. For example, IPV staff training is twice as prevalent in the finance sector as it is in the wholesale and retail sector, paid domestic violence leave is twice as common in the transport/communications sector than it is in any other sector, and financial support to employees is three times as likely to be available in finance as it is in manufacturing. This unevenness in corporations’ policies and programs suggests that employees experiencing IPV are likely to encounter wide-ranging levels of support from their employer, depending on sector employment.

Table 2 describes how listed corporations’ total number of IPV programs and policies varies over the four years (2016–2019) of WGEA data. To a significant extent, these data mirror the discussion above—on average, corporations in 2016 had between 6 and 7 of the programs and policies identified in Table 1. However, the overall average masks significant variation within the sample. In 2016, around one-third of corporations had 5 or fewer IPV programs or policies in place, and over half had 7 or fewer. Bearing in mind that having an overall policy/strategy and any leave provision would give a corporation a score of 3, this suggests that organizational commitment to IPV is relatively low. At the other end of the spectrum, 10% of listed corporations had 12 or more areas of policy or IPV support in place, suggesting that a small proportion of corporations have highly responsive approaches to IPV. Over time, corporations’ programs are improving significantly—by, on average, around 1 policy area per year—and over the 4-year data period the overall average number of policy/programs in place among sample corporations grew from 6.4 to 9.4. This jump in the implementation of policy and programs is very encouraging and indicates that the availability of the data and the comparative benchmarking that it affords are influencing listed corporations’ efforts to be more responsive to employees experiencing IPV. At the same time, the largest change over this period is at the bottom end of the distribution. For example, whereas almost 30% of corporations had 5 or fewer measures in place in 2016, this percentage had fallen to around 10% by 2019 suggesting that the primary impact of making the data available has been to encourage those listed corporations with no, or minimal, support or policy in relation to IPV to take more substantive action.

**Table 2:** Variation over time in listed corporations’ total number of IPV policies and programs

		Year			
		2016	2017	2018	2019
0	Progams/Policies	11	9	5	4
		6.7%	5.4%	2.9%	2.2%
1	Progams/Policies	5	1	0	0
		3.1%	0.6%	0.0%	0.0%
2	Progams/Policies	9	7	5	1
		5.5%	4.2%	2.9%	0.6%
3	Progams/Policies	11	8	7	5
		6.7%	4.8%	4.0%	2.8%
4	Progams/Policies	10	5	4	2
		6.1%	3.0%	2.3%	1.1%
5	Progams/Policies	16	14	16	12
		9.8%	8.4%	9.2%	6.7%
6	Progams/Policies	24	18	14	14
		14.7%	10.8%	8.0%	7.8%
7	Progams/Policies	17	14	19	15
		10.4%	8.4%	10.9%	8.3%
8	Progams/Policies	15	18	16	21
		9.2%	10.8%	9.2%	11.7%
9	Progams/Policies	18	14	21	20
		11.0%	8.4%	12.1%	11.1%
10	Progams/Policies	8	13	14	16
		4.9%	7.8%	8.0%	8.9%
11	Progams/Policies	3	15	14	18
		1.8%	9.0%	8.0%	10.0%
12	Progams/Policies	6	8	11	13
		3.7%	4.8%	6.3%	7.2%
13	Progams/Policies	6	7	6	14
		3.7%	4.2%	3.4%	7.8%
14	Progams/Policies	2	6	9	9
		1.2%	3.6%	5.2%	5.0%
15	Progams/Policies	2	6	8	9
		1.2%	3.6%	4.6%	5.0%
16	Progams/Policies	0	2	5	6
		0.0%	1.2%	2.9%	3.3%
17	Progams/Policies	0	1	0	1
		0.0%	0.6%	0.0%	0.6%
Total number of firms/year		163	166	174	180
Overall average number of programs/Policies		6.39	7.89	8.57	9.40

### Influences on Listed Corporations’ IPV Responsiveness

Having described the sectoral pattern and change over time in listed corporations’ IPV policies and programs, we turn to modelling the factors associated with corporate IPV responsiveness—as captured by the count of a corporation’s policies and programs present each year—to test our hypotheses. These findings are reported in Table 3. Model 1 establishes a baseline model that includes a corporation’s size, industry sector, and the year as explanatory variables. Building on the descriptive analysis above, Model 1 shows that size, sector, and time matter a lot for IPV responsiveness—bigger corporations are more responsive (P < 0.000), and corporations have significantly more policy and support programs in place in 2017, 2018, and 2019 when compared to 2016 (P < 0.000, P < 0.000, and P < 0.000 respectively). These findings support hypotheses 1, 2, and 3, suggesting that institutional context plays an important role in shaping listed corporations’ IPV responsiveness. Reflecting the possible importance of stakeholder pressures for IPV responsiveness, model 2 examines relationships between workforce composition and IPV responsiveness. Do listed corporations that employ more women exhibit greater IPV responsiveness? Do corporations that have more women in managerial, leadership, or governance positions have better IPV responsiveness? The results in Model 2 show that there is no significant relationship between the overall rate of female employment and corporations’ IPV policies and programs. Thus, a greater intensity of female employees is not, per se, associated with greater IPV responsiveness. At the same time, we identify two statistically significant relationships—both the percentage of a corporation’s managers that are women, and the percentage of a corporation’s directors that are women are found to be highly significantly (P < 0.01, and P < 0.05, respectively) and positively associated with corporations’ overall IPV responsiveness. Overall, this provides mixed evidence in relation to hypothesis 4 in that while we find no evidence of a relationship between corporations’ overall proportion of women employees and IPV responsiveness, we do find relationships with the prevalence of women management and directors. Quantitatively, the association between women managers and IPV provisions is between 4 and 5 times as large as that between IPV provisions and the percentage of women on a corporation’s board. Increasing the proportion of a corporation’s women managers by 10% (from its average of 36%) is, approximately, associated with an increase of 1 in the number of IPV provisions at a corporation. In contrast, increasing the percentage of women on a corporation’s board by 10% (from its average of 26.5%) would lead to an increase of about one quarter of an IPV provision. Overall, our evidence concludes that the percentage of women in a corporation’s middle management tier plays a fundamental role in shaping IPV responsiveness.

**Table 3:** Regression models of the influences on listed corporations’ total number of IPV policies and programs

	Model 1	Model 2	Model 3	Model 4	Model 5	Model 6
*Control variables*						
Constant	− 2.977*** (0.914)	− 3.000*** (0.925)	− 3.874*** (0.982)	− 0.352 (1.135)	− 2.066** (0.915)	− 1.311 (1.169)
SIZE	0.980*** (0.11)	0.846*** (0.111)	0.994*** (0.118)	0.585*** (0.15)	0.737*** (0.117)	0.310** (0.155)
YEAR_2019	2.680*** (0.335)	2.347*** (0.33)	2.672*** (0.334)	2.822*** (0.334)	2.639*** (0.329)	2.432*** (0.319)
YEAR_2018	1.990*** (0.337)	1.702*** (0.33)	1.970*** (0.336)	2.025*** (0.333)	1.961*** (0.33)	1.699*** (0.317)
YEAR_2017	1.243*** (0.34)	1.142*** (0.328)	1.220*** (0.339)	1.282*** (0.337)	1.294*** (0.334)	1.221*** (0.316)
*Workforce composition*						
Overall % female employees		− 0.028 (0.019)				− 0.019 (0.019)
Managers % female		0.108*** (0.022)				0.102*** (0.021)
Senior managers % female		− 0.016 (0.01)				− 0.015 (0.01)
Directors% female		0.022** (0.011)				0.019* (0.011)
*Financial resource availability*						
Long term debt % assets			0.010 (0.011)			0.013 (0.01)
Cash as & Assets			0.036*** (0.012)			0.046*** (0.011)
Return on assets %			− 0.003 (0.016)			0.001 (0.015)
*Corporate visibility*						
Member of ASX50				1.903*** (0.497)		1.392*** (0.47)
*Employee representation*						
Salaries by industrial agreement					− 0.556 (0.511)	− 0.944* (0.487)
Employee consultation on gender equity					1.685*** (0.324)	1.854*** (0.309)
Dummy Variables for 2-Digit SIC Industry Sectors Included	YES	YES	YES	YES	YES	YES
Number of Observations	682	682	682	682	682	682
R-Squared	0.392	0.443	0.401	0.406	0.419	0.494
R-Squared Adjusted	0.335	0.387	0.342	0.350	0.362	0.438

Figures in parentheses are standard errors; *p < 0.10; **p < 0.05; ***p < 0.01

Model 3 explores the role of corporations’ financial resources on IPV responsiveness, reflecting the possible importance of care-based normative stakeholder concerns for IPV responsiveness. Early research on social responsibility programmes, and research on flexible working, employee health and safety and other employee-directed programs suggests that the availability of discretionary financial resources might play an important role in enabling more responsive policies to be implemented. Our findings imply that this might also be the case for IPV provisions in that corporations with more abundant cash resources are found to have better IPV responsiveness (P < 0.01). However, we find no significant relationship between either profitability or debt and IPV responsiveness, indicating that not all forms of financial resource contribute equally to encouraging IPV responsiveness. Thus, we discover mixed evidence in relation to hypothesis 6. Model 4 investigates the relationship between membership of the most prominent group of Australian listed corporations—the ASX50—and IPV responsiveness. Prior research concluded that visibility/prominence in each context can play an important role in encouraging corporations to exhibit responsiveness to stakeholder needs. Thus, there is strong evidence that members of the ASX50 have significantly higher levels of IPV responsiveness than other corporations (P < 0.01), with ASX50 listed corporations having on average 2 more areas of active IPV policy or program than other corporations, ceteris paribus. This finding is further evidence in support of hypothesis 1.

Model 5 examines the possible importance of employee representation and consultation for IPV responsiveness. Although no significant relationship between salaries being set by industrial agreement (a proxy for union involvement) and IPV responsiveness can be concluded, a strongly positive association between employee consultation and IPV responsiveness can be identified—statistically significant at the 1% level (P < 0.01) and in magnitude equivalent to an improvement of more than 1.5 policy/program initiatives. Therefore, listed corporations that formally engage and consult with their employees on gender equity are likely to have significantly better IPV responsiveness, consistent with hypothesis 5.

Finally, Model 6 combines all our previous models to determine which attributes and conditions are, overall and when controlling for all the other factors, most strongly associated with better IPV responsiveness. Generally, the findings mirror those described above in that IPV responsiveness is highest among larger corporations, especially members of the ASX50, in more recent years, in settings where there is a higher proportion of women managers, where corporations have more funds available, and where there is formal employee consultation on gender equity.

## Discussion

The descriptive analysis of listed corporations’ responses to IPV suggests that corporations have a long way to go to support employees experiencing IPV. Overall, evidence suggests that IPV responses are on average relatively under-developed and highly variable across industry sectors, though they are improving consistently over time. Most corporations analysed in the study have relatively shallow implementation of IPV responsiveness measures, policies, and practices. However, support for IPV is changing, and quite rapidly. The regression analysis of corporate IPV responsiveness suggests that listed corporations’ IPV supports reflect institutional pressures as well as a desire to address stakeholder concerns, both because doing so is in corporations’ self-interest, and because of a normative concern for employees experiencing IPV. As expected, those listed corporations with increased resources including financial resources are more pro-active and provide greater support. However, our findings also suggest several other key influences on corporate IPV responsiveness.

**Fig. 2:**
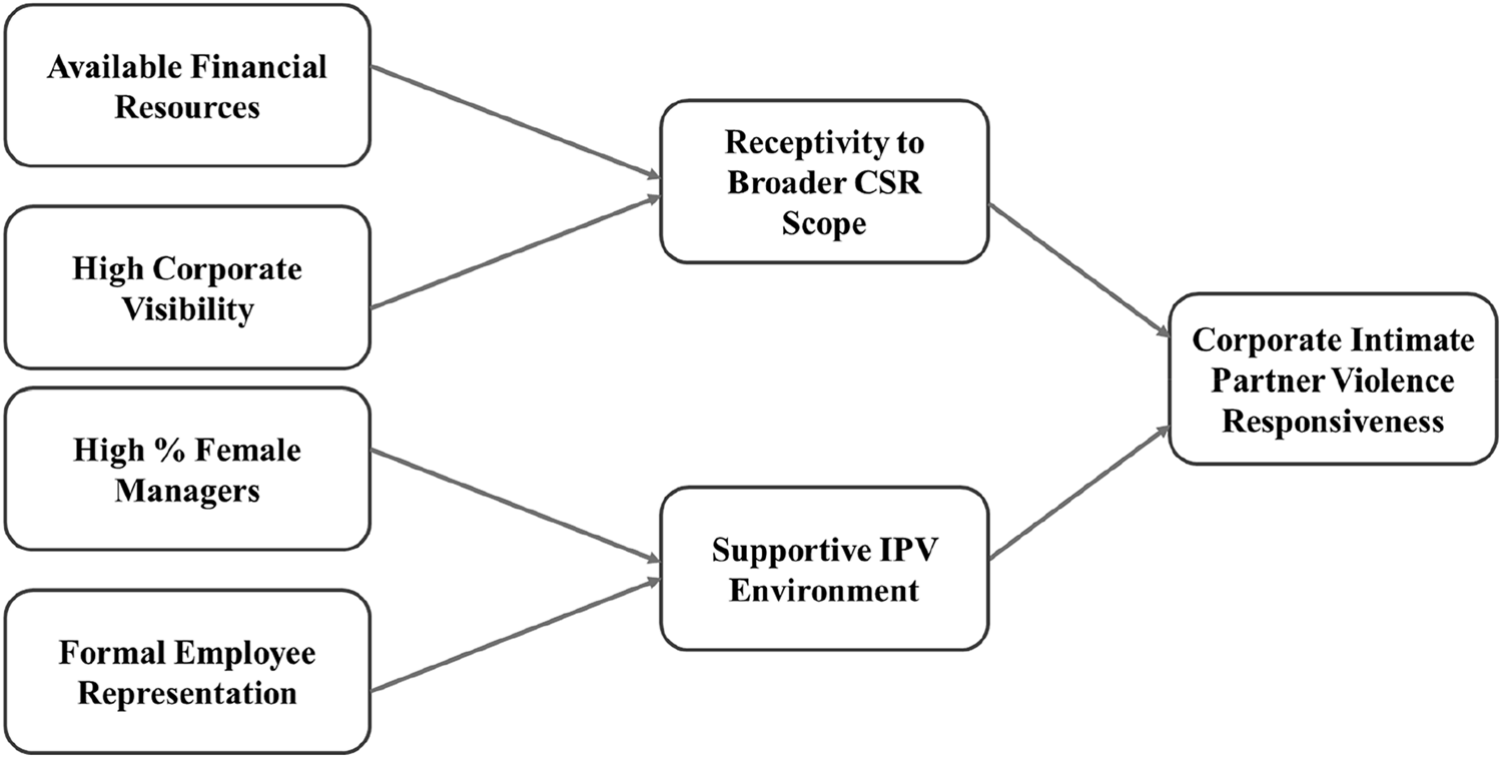
Synthesis of key findings to conceptualise influences on corporate IPV responsiveness

Figure 2 synthesises our strongest findings into an overall conceptual model that identifies four key relationships that influence listed corporations’ IPV responsiveness. Additionally, building on prior literature, we propose two possible intermediary processes, receptivity to broader CSR scope and supportive IPV environment, to help to explain our key findings.

Our first cluster of key findings suggests that corporate visibility and the availability of financial resources, are strongly associated with IPV responsiveness. Building on prior literature, these factors influence corporations’ receptivity and openness to accepting broader social responsibilities (see, e.g., Durand et al., 2019; Parker et al., 2019; Wiengarten et al., 2017). Prior research has emphasised that corporate willingness to adopt new CSR practices is especially heightened in circumstances where responses are highly visible to stakeholders (Tetrault-Sirsly & Lamertz, 2008), and where the issues concerned are highly salient to stakeholders (Durand et al., 2019), both of which apply in the context of IPV responsiveness in Australia. Therefore, IPV responsiveness has strategic importance in that it positions listed corporations externally in relation to peers and in relation to concerns regarding fostering greater equality and inclusion. For decades, corporate diversity management strategies have been charged with window-dressing and pink-washing gender equality efforts. For this reason, whilst corporate IPV responses go some way to incorporating concern for equality and inclusion within CSR, organizational responsibility for IPV responsiveness needs further commitment.

Regarding our second cluster of key findings, we conclude that both higher percentages of female managers and the presence of formal employee consultation on gender equity through formal employee representation are associated with greater IPV responsiveness. Building on prior research (Laharnar et al., 2015; MacGregor et al., 2021; Swanberg et al., 2007; Swanberg & Logan, 2005), we propose that these factors reflect the supportiveness of a corporation’s IPV environment through organizational programmes and policies (Swanberg et al., 2007) and workplace interventions (Adhia et al., 2019).

Our findings suggest that IPV responsiveness is positively associated with the greater prevalence of female middle managers within a corporate setting. While our analysis is unable to test how female managers affect corporate IPV responsiveness, one explanation for the importance of female managers in our study may relate to the gendered nature of IPV experiences and the importance of informal, culturally sensitive pathways to appropriately and safely supporting employees who choose to disclose IPV. Prior research has suggested that employees experiencing IPV can face a type of “double violence” whereby workplace performance management practices introduce a second layer of violence to employees’ experience of IPV which compound the negative effects of, for example, work interference (Al-Modallal et al., 2016). One suggestion for avoiding ‘double violence’ is to develop culturally sensitive work environments where it becomes feasible and safe for employees to disclose IPV (Bennett et al., 2021; Swanberg & Logan, 2005). Yet, from an ethical perspective, corporate IPV responses predicated on employee disclosure of IPV risk being insufficiently inclusive and comprehensive in their scope (Bennett et al., 2021). Many employees remain reluctant, or even unable, to disclose IPV due to fears for personal safety, experiences of shame, stigma, or indignity, or concerns about the detrimental effects of disclosure on job continuity or progression (Al-Modallal et al., 2016; MacGregor et al., 2021, 2022). Additionally, individual working conditions may not be conducive to IPV disclosure because of a poor relationship between the employee and manager, a manager’s discomfort, or inexperience with IPV (Adhia et al., 2019; MacGregor et al., 2022; MacQuarrie et al., 2019), or because of individualistic, hyper-masculine and high-performance work cultures. We therefore propose that the significant barriers to disclosure must be accounted for in the design of IPV policies and practices.

Our findings suggest that corporations’ industrial relations climate—as reflected in the presence of formalized employee consultative mechanisms and dialogue opportunities regarding gender equity—play an important role in sensitising corporations to their responsibility for IPV and associated concerns regardless of disclosure. In line with previous work (for example, Bennett et al., 2021; Gracia et al., 2020; Wibberley et al., 2018) we therefore propose that generalised IPV sensitivity and supports that transcend individual disclosure may be important, because ethically it should not be essential for an employee to disclose IPV at work to be supported in various ways. Such supports may include safety mechanisms that are accessible to all employees (e.g., ‘no questions asked’ leave) (Adhia et al., 2019; Glass et al., 2016), Employee Assistance Programmes (e.g., focussing on any personal problem that may affect work performance) (Adhia et al., 2019; Bennett et al., 2021; Falk et al., 2001; MacGregor et al., 2021), training and education for all employees about IPV (Bennett et al., 2021; MacGregor et al., 2017), and access to resources outside the workplace (Adhia et al., 2019; Navarro et al., 2014; Showalter et al., 2021).

Where industrial relations conditions provide for periodic formal dialogues between employee representatives and employers, we find significantly better IPV responsiveness, suggesting that these dialogue mechanisms contribute to the creation of a greater level of generalised responsiveness towards employees on issues such as gender equity initiatives, that contribute to an environment which addresses IPV when it is disclosed. It might be that the routines associated with these employer-employee dialogues help to build understanding and concern among employers. A proactive approach that considers IPV as a structural and cultural issue would advance organizations’ position in taking a progressive stance on IPV, focusing on responsibility for, rather than just responsiveness, to IPV.

### Implications for Policy and Practice

The findings presented in this paper have several important implications for policy and practice. Australia’s approach to addressing gender inequities perpetuated by IPV has so far emphasized mandatory reporting by organizations, involving an implicit assumption that transparent public reporting will encourage organizations to improve practices without the need to introduce new regulation and legislation. Our evidence suggests that even though corporate IPV responsiveness is increasing over time among Australian listed corporations, current responses are, on average, modest in degree, and confined to a sub-set of large, highly visible, and resource-abundant corporations. Policy makers need to consider whether sufficient progress is being made on IPV and whether mandated reporting goes far enough to encourage listed corporations to develop systematic, fully integrated approaches to responsibly managing IPV. Corporate responsibility can only be achieved if organizations shift their responses from the management of employee experience of IPV to strategic action which takes seriously the structural and cultural changes required to fully integrate responsibility for IPV into organizations.

Current circumstances—both the climate crisis, and especially the Covid-19 pandemic—are contributing to escalating IPV in Australia and elsewhere (Kennedy, 2020; Sharma & Borah, 2020; Usher et al., 2020), confirming Boddy and Harris’s (2021) finding that IPV surges after natural disasters (i.e., bushfires and floods) because the latter fuel unemployment and impact other health and lifestyle factors (Kennedy, 2020). Covid-19 has led to profound changes in patterns of work that have important implications for both IPV and CSR (Parry & Gordon, 2021). More than ever, employees are working from home, elevating concern that homeworking will lead to increasing violence against women (Agüero, 2021; Boxall, Morgan and Brown, 2020; Every-Palmer et al., 2020; Galloway, 2020; Van Gelder et al., 2020). If the current health, climate, and economic crises continue, it should be expected that more people will experience IPV (Boddy & Harris, 2021), requiring significant and urgent policy and organizational innovation and action. IPV has significant long-term impacts on women’s economic opportunities and poverty in later life. Because IPV has wide ranging and long lasting economic and social impacts on women’s families, employment, and communities, greater organizational support for employees experiencing IPV will generate significant positive impacts on gender equity, for example in relation to pay and retirement gaps. Building on the special issue call, recent times have witnessed continued erosion of work and home boundaries (de Jonge, 2018), and have collapsed during Covid-19 provoked by homeworking and home-schooling (Agüero, 2021; Sharma & Borah, 2020). Therefore, organizations require a crisis plan to ensure that these eroding boundaries, are appropriately navigated. This could, for example, be done through shifts in working arrangements, whether in the case of transitioning back into work physically or in adjusting to work remotely.

Alongside concerns to increase receptivity and concern for IPV among listed corporations, our findings highlight some important issues regarding the implementation of IPV initiatives. Findings also indicate the importance of female middle managers and formal employee consultation to IPV responsiveness. Both factors suggest IPV disclosure environment is critical to improved IPV responsiveness. Moreover, there is a challenging tension within organizations between being comprehensive and inclusive in approach, while not seeking to implement a ‘one size fits all approach’ (MacGregor et al., 2022). Given that businesses are important social actors (cf. de Jonge, 2018), we recommend the development of proactive and normative agendas. Organizations are sites which could develop, build, and maintain gender just environments, but they require cultural change to address the prevalence of the gendered organization which favours men and problematic types of masculinity (Acker, 1990; Wilcox, et al. 2021) and which often normalizes violence. In this process, we propose that developing integrated, supportive, and inclusive approaches to IPV disclosure is especially important. Employees experiencing IPV find disclosure to organizational actors highly uncomfortable, and IPV typically presents indirectly via performance issues within the workplace (Wibberley et al., 2018). A supportive IPV disclosure environment therefore prevents double violence in which the primary violence of IPV is unintentionally compounded by organizational performance processes and workplace exclusion.

Given its gendered nature, IPV should be an integrated component of gender equality strategies and policies in all organizations. An important start to ensure a sustained responsible rather than a reactive approach would be to embed IPV into a CSR approach for managing people, from focusing on the areas of job security, paid-leave, career planning to receiving everyday welfare, legal or childcare support, mental health support, and other specialist advice. All these process and acts demonstrate that employers are able to perform their duty of care. This duty of care can only be implemented if responsibility for IPV is rolled out to all levels and areas of the organization, and this approach relies on providing training on responsibility and responsiveness for IPV to managers with direct reports and other key staff, while acknowledging the challenges of implementing such training (Rodriguez et al., 2021). To advance organizational responsiveness and responsibility, with the focus of eradicating inequality (Simonovic, 2020), careful consideration of the diversity of women facing IPV is required to respond to intersectional differences in culturally sensitive ways. Moreover, considering IPV in the context of the intersections between social and organizational marginalization and discrimination of women and gender-based violence (Dlamini, 2021) is critical. Putting the diversity of women facing IPV and their lived experiences at the centre of developing organizational approaches will ensure that structural and cultural organizational problems which perpetuate and sustain inequalities surrounding IPV are implemented. Interventions most probably need to be led by expert women who understand the complexities and who are in positions to hold organizations accountable. Organizations need to design and implement supports that holds them accountable to the increasing demands of IPV. Building organizations which educate the risks and dangers of gender-based violent behaviours are overdue, as are responsible management which facilitate women’s economic equity. These recommendations would probably need closer partnerships between government, civil organizations, and business organizations.

### Limitations and Implications for Future Research

This study has three key limitations that highlight future avenues for research. First, unlike the study by MacGregor et al. (2017) on resources for domestic violence in the Canadian workplace, our study focuses on organizational reporting of their own IPV policies and practices. Extending this study to consider employee experiences of organizational responsiveness in the Australian context would be a useful step in understanding gaps that exist between the espoused organizational commitment to IPV and the lived employee experiences of IPV policies. The focus has also been with publicly listed corporations, and it would be useful to compare the IPV responsiveness of these with SMEs and public/third sector organizations. Second, it is important to note that the two intermediary processes, receptivity to broader CSR scope and supportive IPV environment, suggested in our proposed model have not been tested in this study. For example, while we develop some suggestions from the literature, we are unable to demonstrate from our quantitative data the mechanisms by which a higher proportion of female managers contributes to corporate IPV responsiveness. Future research could therefore usefully explore the intermediary processes that mediate the relationships between organizational characteristics and corporate IPV responsiveness. Third, our study focuses on a single country context. Organizations operating in Australia experience relatively high levels of normative institutional pressure (e.g., from Women’s Safety New South Wales), with the relationship between IPV and work being “… structured through relations of power beyond gender” (Farhall, 2020, p.105), such as the enduring effects of settler colonialism on Indigenous in/equality and the reduced visibility of IPV in indigenous and rural communities. As noted in the special issue call, our research, like much of the extant IPV research, is situated in a developed “Anglo-American”, liberal market economy institutional context. Given the likely variations that stem from institutional influences and gender equity concerns, further research is needed across geographical regions and country contexts, especially focusing on IPV in less well understood institutional contexts. For example, Pachner et al. (2021) call for specific workplace support for Black women survivors of IPV in the United States after workplace disruptions and by Parry and Gordon (2021) who illustrate the impact of COVID-19 in South Africa given the precarity of women’s lives and access to employment.

We support calls for further research into corporate responses to IPV especially research that explores the tensions between addressing IPV from a responsible human resource management perspective and exploring the business case whether it is lowering costs associated with IPV, increasing employee performance, or managing risks to other employees. In addition, researching the complexities surrounding the welfare of employees and variations in employee experiences (i.e., gender, sexual orientation, role, tenure etc.) would enable human-centred approaches to the research. Moreover, thinking through the intersection between the design of structural interventions and the dynamics between IPV reporting and support at local levels would enable responsibility to shift towards the organization instead of the employees experiencing IPV. If organizations fully understand the risks and costs to their business, proactive, systematic approaches to IPV that are contingent on sector, organizational and cultural contexts could be designed and maintained.

Future research could build on feminist approaches to CSR (Grosser & Tyler, 2022) to consider IPV in the context of human rights approaches to gender-based violence. A human rights approach to addressing IPV in organizations would ensure a responsible, sustained and just approach—and it would put women’s diverse experiences of marginalisation and discrimination at the heart of improving responsibility for IPV in organizations. Extensive research and funding are required to understand the local contingencies of women facing IPV across geopolitical contexts.

## Conclusion

This study provides unique insights into the influences on corporate policies and support mechanisms available in relation to IPV in Australian listed corporations between 2016 and 2019. IPV is among global society’s most pernicious and impactful issues, causing substantial harm to women’s health and wellbeing, and generating significant lost income and productivity. Yet, in contrast to other employee- and gender-related social issues, very little business ethics research has explored corporate IPV responsiveness. In this paper we have provided the first large-scale empirical analysis of corporate IPV policies and practices, illuminating both overall trends in how listed corporations are supporting employees experiencing IPV and patterns of variation across corporations in IPV responsiveness. Our findings identify greater IPV responsiveness among larger listed corporations, as well as those with a higher proportion of women middle managers, greater slack resources, and better formal employee consultation on gender issues. Surprisingly, we find no association between IPV responsiveness and the overall proportion of women in corporations’ workforce and boards of directors. We conclude that a mixture of instrumental and institutional factors appears to best explain patterns in IPV responsiveness and propose that there are significant opportunities for organizations in developing a human centric, care-based approach to IPV, an approach which would put socially responsible corporate practices to work to reduce violence for women employees.

## Footnotes

1 The WGEA Data Explorer is available at: https://data.wgea.gov.au/organisations

2 The complete WGEA organization questionnaire is available at: https://www.wgea.gov.au/what-we-do/reporting
